# Perioperative bronchoaspiration in a semaglutide user on a residue-free diet: a case report and insights from a complication

**DOI:** 10.1186/s13741-025-00603-y

**Published:** 2025-10-24

**Authors:** Leonardo Barbosa Santos, Leopoldo Muniz da Silva, Saullo Q. Silveira, Rafael S. F. Nersessian, Giulia D. Matheus, Glenio B. Mizubuti, Joaquim Edson Vieira

**Affiliations:** 1 Surgical Sciences and Perioperative Medicine, University of Sao Paulo, Postgraduate Program in Anesthesiology, São Paulo, Brazil; 2 CMA Anesthesia Group, Vila Nova Star Hospital, São Paulo, Brazil; 3 D’Or Institute for Research and Education, São Paulo, Brazil; 4 Nove de Julho University, São Paulo, Brazil; 5 Department of Anesthesiology and Perioperative Medicine, Queen’s University, Kingston, Canada

**Keywords:** Bronchoaspiration, Glucagon-like peptide-1 receptor agonist, Patient safety, Preoperative liquid diet, Semaglutide

## Abstract

**Introduction:**

Perioperative bronchoaspiration is a serious complication often associated with inadequate fasting or delayed gastric emptying, including that caused by glucagon-like peptide-1 receptor agonists (GLP-1-RAs). Despite growing semaglutide use worldwide, evidence on the effectiveness of current preventive measures—such as residue-free diets—remains limited.

**Case presentation:**

We report a 61-year-old female with obesity and chronic obstructive pulmonary disease who underwent elective coronary angiography. She had been using weekly semaglutide for weight loss, discontinued six days before the procedure, but did not disclose this during preoperative evaluation. Following institutional guidance, she adhered to a 24-h residue-free diet and 12-h fasting. A protocol breach led to omission of preoperative gastric ultrasound. During anesthesia induction, she experienced large-volume regurgitation requiring urgent airway management. Postoperative chest CT revealed aspiration-related inflammatory changes. She recovered uneventfully and later acknowledged omitting semaglutide use from her medical history because she did not consider it a “medication.”

**Conclusion:**

This case demonstrates that even stricter dietary measures than those recommended in current guidelines may not eliminate aspiration risk in GLP-1-RA users. Active screening for GLP-1-RA use, consideration of extended discontinuation intervals, and routine bedside gastric ultrasound should be incorporated into perioperative protocols to enhance patient safety.

## Introduction

Perioperative bronchoaspiration is a life-threatening complication, frequently associated with failures in preoperative fasting and/or conditions/medications (e.g., glucagon-like peptide-1 receptor agonists—GLP-1-RAs) that delay gastric emptying (Warner et al. 1993). With the rapid increase in semaglutide use worldwide, anesthesiologists must remain vigilant about its associated risks, particularly the potential for perioperative bronchoaspiration (Santos et al. 2024a). Evidence indicates that GLP-1-RA users present more often with increased residual gastric content—a key mediator of aspiration risk—compared with non-users: 56% vs. 19% on preprocedural gastric ultrasonography in a cross-sectional study (Sen et al. 2024) and 19% vs. 5% in a historical endoscopy cohort, in which one aspiration event was reported (Wu et al. 2024).

Recently, a multi-society consensus was published which, although not based on robust evidence, represents the most current guidance in the literature, offering recommendations for the perioperative management of GLP-1-RA users. For patients without risk factors, therapy continuation is recommended. In high-risk cases, a 24-h liquid diet preoperatively is suggested to reduce gastric retention. In select situations, preoperative GLP-1-RA discontinuation may be considered, with the caveat that it may result in perioperative hyperglycemia. Other mitigating strategies against perioperative bronchoaspiration include rapid sequence induction and intubation and the use of bedside gastric ultrasound to assess residual gastric contents prior to anesthesia induction, balancing therapy continuation with aspiration risk minimization (Kindel et al. 2024).

**Fig. 1:**
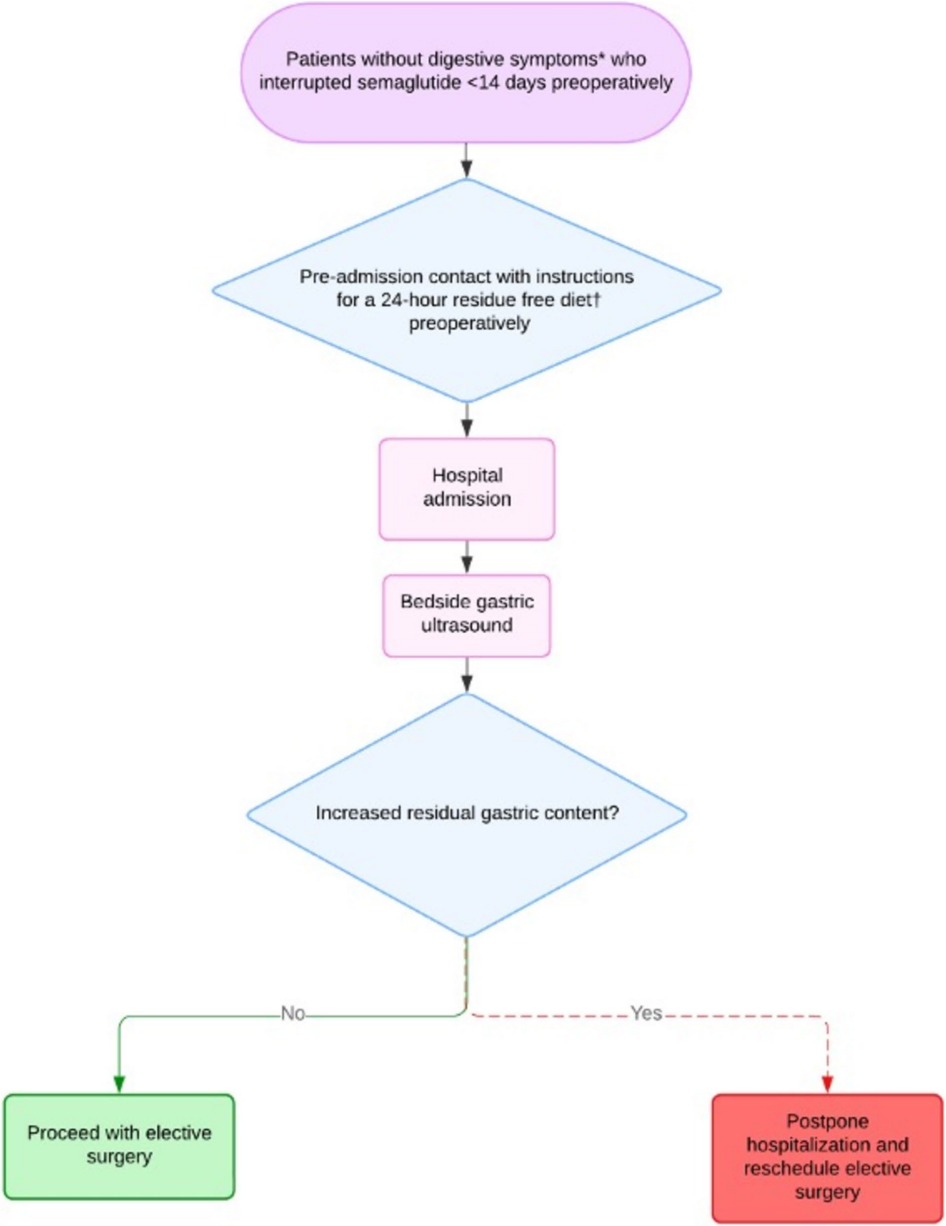
Institutional protocol for perioperative management of patients on weekly subcutaneous semaglutide undergoing elective surgical procedures under anesthesia. *Digestive symptoms: nausea/vomiting, dyspepsia, and/or bloating. †Residue-free diet: consists of easily digestible foods that leave no residue in the digestive tract, including French bread, water and salt crackers, boiled or poached eggs, white rice, plain pasta without sauce, strained vegetable or meat broths, UHT juices (grape, peach, or apple) with no added sugar and no residue, coffee, fruit teas, coconut water, gelatin (sugar-free for diabetic patients), and fruit popsicles without milk or fruit pieces. Foods such as dairy products, meats (except when strained in broth), whole grains, raw fruits and vegetables, spices, and fried foods must be avoided. The recommended preoperative fasting period is 8 h for both solids and liquids

Our group recently conducted a study (Santos et al. 2024) in which discontinuing semaglutide for more than 21 days preoperatively in patients with digestive symptoms, and more than 14 days in asymptomatic patients, resulted in gastric retention rates comparable to those of non-users. Based on these findings, we have implemented, in collaboration with surgical teams, an institutional protocol (Fig. 1) that includes specific recommendations for semaglutide discontinuation intervals, a residue-free diet, and preoperative fasting.

This case underscores that adherence to a preoperative residue-free diet, although recommended to minimize gastric retention in GLP-1-RA users, may not reliably ensure complete gastric emptying. Furthermore, protocol deviations—such as omission of gastric ultrasound—can contribute to adverse events. We present this report to highlight a critical gap in current perioperative management recommendations: the persistence of aspiration risk despite compliance with dietary measures. This observation reinforces previous calls for active screening for GLP-1-RA use during the pre-anesthetic evaluation^2^ and systematic gastric content assessment as integral components of perioperative protocols, as well as the need to refine current guidelines to mitigate this emerging risk.

## Case report

Six days before the procedure: a 61-year-old female with significant comorbidities, including a 30 pack-year smoking history, chronic obstructive pulmonary disease, obesity (body mass index: 34.9 kg·m⁻^2^), and dyslipidemia, with no relevant family history, was scheduled for elective coronary angiography. She had been using subcutaneous semaglutide (1 mg weekly) for weight loss over the preceding four months, with the last dose administered six days before the procedure.

Five days before the procedure: the patient was contacted through an electronic survey (chatbot) routinely provided to all surgical candidates regarding semaglutide use. She reported a discontinuation interval of six days before the procedure. In cases where the interval is less than 15 days before surgery (Santos et al. 2024b), a healthcare professional contacts the patient and provides guidance on a residue-free diet.

Twenty-four hours before the procedure: following this guidance, she adhered to a strict 24-h residue-free diet as recommended by the institutional protocol (Fig. 1).

Twelve hours before the procedure: she complied with nil per os for both solids and liquids.

Day of procedure – pre-anesthetic evaluation: upon hospital admission, she was questioned about her continuous medications, which included sertraline, quetiapine, lamotrigine, and methylphenidate. There were no comorbidities associated with delayed gastric emptying. However, she did not report recent semaglutide use within the last six days, later explaining that she did not consider it a “medication” since it was taken for aesthetic weight loss purposes.

Day of procedure – protocol breach: according to the institutional protocol (Fig. 1), bedside gastric ultrasound should have been performed to assess residual gastric content on the day of admission. Due to a protocol breach, this examination was not performed, and the patient proceeded to the operating room.

Day of procedure – anesthesia induction: upon initial sedation, the patient exhibited large-volume regurgitation of yellow liquid content. The attending anesthesiologist immediately suctioned the airway and proceeded with urgent rapid sequence tracheal intubation. Upon direct laryngoscopy, no material was observed entering the glottis; however, a small amount of gastric content was retrieved upon active suctioning of the endotracheal tube. Intraoperative bronchoscopy was not performed.

Postoperative day 0: in the post-anesthetic care unit, her respiratory status was closely monitored. At the request of the surgical team, empirical clindamycin was initiated to prevent secondary pulmonary infection. Pulse oximetry (SpO₂) remained 84–88% on room air, requiring nasal oxygen at 2 L/min to maintain SpO₂ ≥ 92%. She remained eupneic, and pulmonary auscultation was unremarkable.

Postoperative day 1: the patient reported only a mild dry cough. No corticosteroids were administered. A chest computed tomography scan revealed centrilobular opacities and ground-glass infiltrates, suggestive of aspiration-induced inflammation. Nasal oxygen was discontinued by the end of the day.

Postoperative day 3: she was discharged without sequelae or symptoms. Upon further questioning, she disclosed semaglutide use (1 mg subcutaneously) six days before the procedure—a detail omitted during pre-anesthetic evaluation. She confirmed weekly use for four months and reiterated that she did not consider it a “medication.” Family members corroborated that she adhered to the 24-h residue-free diet and 12-h nil per os period as per protocol.

Written informed consent for publication of this case and any accompanying images was obtained from the patient.

## Discussion

This case raises questions regarding the true perioperative benefit of restrictive diets—such as residue-free regimens—for GLP-1 receptor agonist (GLP-1-RA) users. Although current consensus guidelines recommend a 24-h liquid diet in high-risk patients to reduce gastric retention, our institution applies a stricter residue-free protocol aiming to further minimize aspiration risk. Nevertheless, this case demonstrates that even with stricter dietary measures, aspiration may still occur.

Two main factors contributed to this adverse event: the lack of a thorough investigation into semaglutide use during pre-anesthetic evaluation and the absence of gastric ultrasound to assess residual content. Preoperative gastric ultrasonography could have identified retained gastric material, allowing more accurate risk stratification and adoption of additional preventive measures. Even with established institutional protocols, breaches can lead to adverse patient outcomes.

Prior knowledge of GLP-1-RA use would have been crucial for preparing anesthesia induction in a full-stomach patient. Potential strategies could have included pharmacologic agents promoting gastric emptying (e.g., metoclopramide or domperidone), positioning in the reverse Trendelenburg position, rapid sequence induction, and application of the Sellick maneuver (Nason 2015; Dunham et al. 2017; Frerk et al. 2015).

In this case, the patient presented only a dry cough on the first day of hospitalization. According to Warner et al. (1993), patients with clinically apparent aspiration who remain asymptomatic for two hours are unlikely to develop respiratory sequelae; in their cohort, 64% of survivors showed no clinical signs within this period. The use of corticosteroids in this setting remains controversial and has not shown clinical benefit (Janda et al. 2006). Some authors even associate corticosteroid use in gastric aspiration with increased incidence of gram-negative bacillary pneumonia (Wolfe et al. 1977).

Regarding prophylactic antibiotics, their routine use is increasingly discouraged due to the risk of selecting resistant strains (Bynum and Pierce 1976). Current evidence supports therapeutic antibiotic administration based on bronchoalveolar lavage cultures obtained via bronchoscopy (Marik 2001). In this case, bronchoscopy was unavailable, and clindamycin was administered empirically at the surgical team’s request to minimize the risk of secondary bacterial pneumonia. It is important to note that prophylactic antibiotics are not recommended for chemical pneumonitis following witnessed aspiration, as the initial injury is inflammatory rather than infectious (Marik 2001). When aspiration pneumonia is suspected, guidelines suggest beta-lactam/β-lactamase inhibitor regimens (e.g., ampicillin-sulbactam), reserving clindamycin for patients with β-lactam allergy or for classic anaerobic infection syndromes such as lung abscess, necrotizing pneumonia, or empyema (Simpson et al. 2023).

A recent study by Megahed et al. evaluated the clinical impact of early bronchoscopy—within the first 24 h after bronchoaspiration—in mechanically ventilated patients, aiming to remove gastric fluid and solid particles (Megahed et al. 2021). Early bronchoscopy was associated with a significant reduction in pneumonia incidence (60.5% vs. 81.6% in the control group) and improved parameters including hypoxia index, white blood cell count, pulmonary infection score, lung injury score, and Sequential Organ Failure Assessment (SOFA) score (Megahed et al. 2021).

## Strengths, limitations, and future directions

This case provides relevant clinical insight into the perioperative risks associated with GLP-1-RA use, particularly semaglutide, even when stricter preventive measures are implemented. It highlights real-world protocol deviations—specifically, omission of gastric ultrasound—that can occur despite institutional guidelines, offering practical lessons for anesthetic safety. The detailed chronological account, combined with imaging and clinical outcomes, enhances its educational value.

However, as a single case report, generalizability is inherently limited. Causality between semaglutide use, dietary measures, and aspiration risk cannot be firmly established. Furthermore, the absence of confirmatory gastric content assessment (e.g., gastric ultrasound or intraoperative bronchoscopy) limits objective quantification of residual gastric volume and content characteristics.

Future research should aim to define the optimal perioperative discontinuation interval for GLP-1-RA users, stratified by symptomatology, and to evaluate the efficacy of preoperative gastric ultrasound in detecting residual gastric content and reducing aspiration risk. Additionally, studies assessing multi-barrier strategies—combining dietary modification, prokinetic agents, and standardized airway management—may contribute to safer perioperative care in this growing patient population.

## Conclusion

This case underscores the importance of a comprehensive perioperative evaluation in GLP-1-RA users, extending beyond diet and fasting guidelines. While a residue-free diet may reduce the risk of gastric retention, it should not be considered an absolute protective measure against aspiration in patients with delayed gastric emptying. Active screening for medications that impair gastric emptying, combined with gastric ultrasound, should be incorporated as complementary strategies to enhance patient safety. Furthermore, institutional adoption of structured, multi-barrier approaches—including dietary modifications, pharmacologic interventions, and standardized airway management—may help prevent such complications and optimize perioperative care for this growing patient population.

### Patient perspective

Following discharge, the patient expressed satisfaction with the care received and acknowledged the seriousness of the intraoperative event. She reported that the experience increased her awareness of the importance of disclosing all medications—including those used for non-medical purposes—during preoperative evaluation. She also stated that the rapid response of the medical team and her complete recovery without complications reinforced her trust in the healthcare system.

## Data Availability

No datasets were generated or analysed during the current study.
